# A Computer Vision-Based Navigation and Localization Method for Station-Moving Aircraft Transport Platform with Dual Cameras

**DOI:** 10.3390/s20010279

**Published:** 2020-01-03

**Authors:** Jianming Tang, Weidong Zhu, Yunbo Bi

**Affiliations:** 1 State Key Laboratory of Fluid Power and Mechatronic System, College of Mechanical Engineering, Zhejiang University, Hangzhou 310027, China; tang_jm@zju.edu.cn (J.T.); zwdpub@126.com (W.Z.); 2 Key Laboratory of Advanced Manufacturing Technology of Zhejiang Province, College of Mechanical Engineering, Zhejiang University, Hangzhou 310027, China

**Keywords:** aircraft final assembly, vision navigation, transport equipment, CMOS sensor, localization method

## Abstract

In order to develop equipment adapted to the aircraft pulse final assembly line, a vision-based aircraft transport platform system is developed. This article explores a guiding method between assembly stations which is low-cost and easy to change routes by using two-dimensional code and two complementary metal oxide semiconductor (CMOS) cameras. The two cameras installed on the front and back of the platform read the two-dimensional code containing station information to guide the platform. In the process of guiding, the theoretical position and posture of the platform at each assembly station are known, but there is a difference between the actual and theoretical values due to motion errors. To reduce the influence of the deviation on the navigation route, a localization method is proposed based on the two-dimensional images captured by the cameras. Canny edge detection is applied to the processed image to obtain the position of the two-dimensional code in the image, which can measure the angle/distance deviation of the platform. Then, the computer can locate the platform precisely by the information in the two-dimensional code and the deviation measured by the image. To verify the feasibility of the proposed method, experiments have been performed on the developed platform system. The results show that the distance and angle errors of the platform are within ±10 mm and ±0.15° respectively.

## 1. Introduction

In aircraft manufacturing, the assembly has great impact on the production cost, which is estimated to account for almost 30% of the total cost [1,2]. To reduce the manufacturing cost, many aviation companies have introduced the pulse final assembly line for its high efficiency [3]. The aircraft pulse final assembly line is composed of several different assembly stations which are made up of dozens of jobs. During each takt time, an unassembled aircraft enters from one end of the line and stops at the first assembly station. After completing the corresponding assembly jobs at a station, the aircraft is required to move to the next station.

There are two ways to transport the aircraft between the assembly stations [4,5]. The first one is based on the overhead cranes. The aircraft is hoisted by a crane and moved to the next station. As it is dangerous and time-consuming, it is gradually replaced by the second way. The second way adopts the rail and positioners. The aircraft is supported by the positioners and can move together along the rail [6,7]. However, it needs to build the rail and it is difficult to change routes once the rail is built. AGVs (Automatic Guided Vehicle) have been used for repeated transportation tasks in factories for decades [8,9]. For small-sized aircraft, like fighters, it can be more flexible to transport by AGVs. This paper aims to develop an automatic guided platform system for the transportation of small-sized aircraft between assembly stations.

In recent years, various types of indoor navigation and localization methods using magnetic, infrared, laser, and vision sensors have been proposed [10,11,12]. Cameras are cheaper and able to provide more information than other sensors. Besides, vision navigation does not require costly transmitters, receivers, and amplifiers to transmit signals. Therefore, vision navigation is adopted in this paper.

Vision navigation technique can be mainly divided into four categories: lane-tracking navigation, landmark-recognizing navigation, navigation using appearance-based matching, and simultaneous localization and mapping (SLAM) [13]. Landmark-recognizing navigation based on artificial landmarks is easy to change routes, and the artificial landmarks are easy to produce, manipulate, and maintain. Jeisung et al. [14] used fiduciary markers with a capital letter or triangle indicating direction in it to guide a vehicle. Kim et al. [15] applied Artificial Neural Networks (ANNs) to recognize letters of the alphabet on the marker to locate the AGV. Petriu et al. [16] proposed a method of recognizing bit codes on the floor to give the AGV direction. Based on the idea of Petriu et al. [16], some studies [17,18,19] used two-dimensional code to guide the vehicle. A two-dimensional code or QR (Quick Response) code is a graphic marker of a two-dimensional matrix which can store position information. In addition, QR code is small, low cost, and easy to implement. Therefore, QR codes are utilized as landmarks for navigation and localization in this paper.

Rostkowska et al. [20] addressed the application of QR codes as landmarks for mobile robot navigation, but this method of displaying big QR codes on every box is not widely used. Luca et al. [21] used QR codes mounted on a ceiling for smart wheelchair navigation. However, the camera will fail to detect the QR code if the ceiling is too high and it requires at least one QR code in the field of view of the camera at any time. Nguyen et al. [22] attached the QR codes on gaps between tiles on a floor. The robot gets position and orientation information from the QR code and follows the gap line to the next QR code. Unfortunately, there is no tiles on the workshop floor and no gap lines to follow.

Moreover, the previous studies did not take the vehicle or robot size into account. The experiments were all carried out on small prototypes. For long-sized vehicle, such as the platform in this article, a small angle deviation will result in a large distance deviation at the end of the vehicle. Also, the angle deviation may be too small to be detected by the QR code image. As a result, the vehicle can stop at the right position but the distance deviation at the end of the vehicle body is large.

In this paper, QR codes are stuck on the floor at each assembly station. Two CMOS (Complementary Metal Oxide Semiconductor) sensor cameras are installed at the front and back of the platform to capture the QR code images. Therefore, the small angle deviation of the platform can be calculated by the images in the two cameras and the length of the platform. In addition, the drive wheels of the platform are equipped with rotary encoders. In this way, the position of the platform can be calculated by the encoder data when the QR code is not in the field view of the camera.

The main contribution of this paper is to provide a new solution for the transportation of small-sized aircraft between stations in the final assembly line. In addition, the work presents a new method for measuring the small angle deviations of long-sized vehicles in QR code navigation.

This paper organization is as follows: Section 2 describes the system architecture of the transport platform and its workflow in the aircraft final assembly line. The navigation mathematical model and localization method are introduced in Section 3. In Section 4, experiments are presented in detail, including analysis and discussion of the results. Finally, Section 5 collects the conclusions of the work.

## 2. Materials

### 2.1. System Architecture

**Figure 1:**
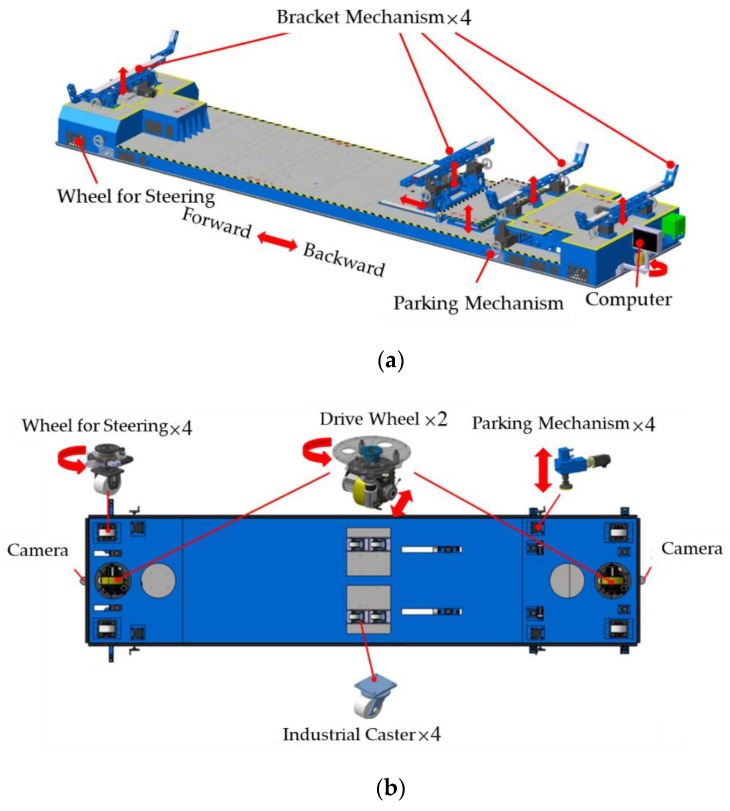
Mechanical structure of the transport platform: (**a**) 3D model; (**b**) bottom view.

The aircraft transport platform is a flexible and numerically controlled system. It consists of a supporting system, a navigation system, and a control system. The mechanical structure of the transport platform is shown in Figure 1, while Table 1 shows the main dimensions and specifications of it.

The supporting system is composed of four parking mechanisms and four bracket mechanisms. The bracket mechanisms are distributed over the transport platform according to the shape and dimensions of the aircraft fuselage, as shown in Figure 1a. They can move up and down singly or synchronously to support the aircraft fuselage. Each bracket contains two pin-axis force sensors, which can monitor the bearing of the bracket mechanism in real time. The parking mechanisms are located at the four corners of the transport platform, as shown in Figure 1b. The supporting foot of the parking mechanism is connected to the screw elevator through a ball head, which can adapt to uneven ground.

The navigation system consists of two drive wheels, four wheels for steering, four industrial casters, and two CMOS cameras. They are used to navigate and locate the platform. The four industrial casters have a swivel design and play an auxiliary supporting role to prevent deformation of the middle part of the platform body. Each drive wheel has two servo motors, which control the forward and backward directions and the rotational direction. Each wheel for steering contains only one servo motor which controls the direction of rotation. The layout of the wheels is shown in Figure 1b. The front and rear CMOS cameras of which the centers are on the central axis of the platform face the floor. A CMOS sensor is a kind of light-emitting pulse sensor, which can convert scene information into a color information matrix for computer processing.

The control system has an industrial computer and an embedded PLC (Programmable Logic Controller CX1020, Beckhoff, Will, Germany), and the data interaction between the two devices is carried out through the TCP/IP communication protocol. The control software is developed with TwinCAT NC (Beckhoff, Will, Germany) and Visual C++ (Microsoft, Redmond, WA, USA). The control of the whole system includes the lifting movement control of the supporting system and the omnidirectional movement control of the platform.

**Figure 2:**
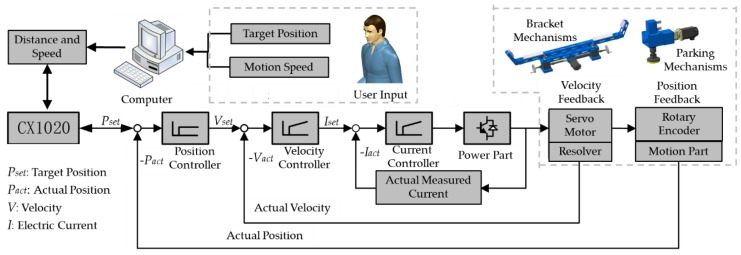
Working principle of the supporting system.

Figure 2 shows the control loop of the supporting system. The speed and position are set by the user through the control software. Then, the computer will generate the motor commands of the movement according to the input and transmit them to the embedded PLC. These commands trigger the servo motors through the drives, generating velocity and moving the bracket mechanisms or parking mechanisms. The servo motors operate in a closed-loop system, which includes three types of control loops: the position loop, the velocity loop, and the current loop. Both the current loop and the velocity loop are PI controllers with proportional and integral gains, while the position loop is a proportional controller. The velocity loop and the current loop are implemented in the servo drives, and the position loop is implemented in the PLC.

**Figure 3:**
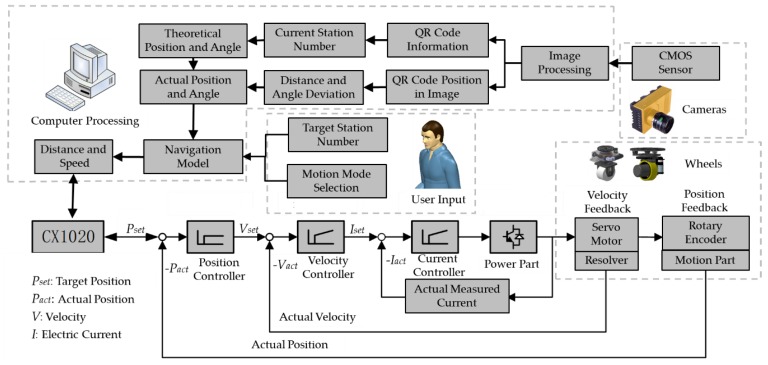
Working principle of the navigation system.

Figure 3 illustrates the navigation control system which needs the user to input a target station number and select a motion mode. The design theoretical position and posture of the aircraft for each assembly station are stored in the computer and are sorted by the station numbers. The station numbers are encoded into QR codes, which are placed on the floor at corresponding stations. After processing the captured QR code image, the position of the QR code in the image is obtained by edge detection, while the station number is obtained by decoding the QR code. Through the proposed localization method, which will be introduced in detail in the next section, the deviation of the platform can be calculated by the QR code images. Then, the actual position and angle can be calculated by the theoretical value and the obtained deviation. Finally, based on the user input and the actual position and angle, the computer calculates the navigation route and generates the servo motor commands. The wheels receive the commands and drive the platform to the target station. In this paper, for the drive motor of the drive wheel, the proportional gain of the position loop is set to 3.5.

### 2.2. Workflow in Aircraft Final Assembly Line

**Figure 4:**
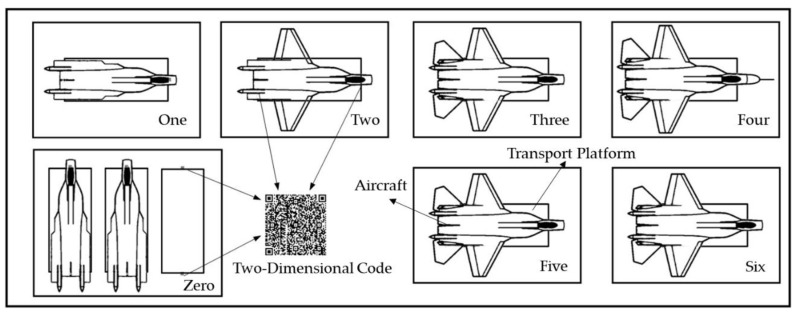
Example of aircraft pulse final assembly line.

As is mentioned in the introduction, the platform is used to transport an aircraft in a pulse final assembly line. Figure 4 shows an example of an aircraft pulse final assembly line. According to the assembly jobs, the production line is divided into seven stations. Station No. 0 is the preparation station, which is used to hoist the fuselage onto the platform and to wait for assembly. The installation of conduits and cables is carried out at No. 1 station. The wings are butted at station No. 2, while the vertical tail is installed at station No. 3. At station No. 4, docking of nose and final test are carried out. Station No. 5 and 6 are used for research and maintenance.

As shown in Figure 4, at each assembly station, QR codes are placed on the floor directly below the cameras of the platform. Then, the transport platform can work in the assembly line, and the specific process is as follows:

(a) Hoist the aircraft onto the platform. Drive the platform below the aircraft fuselage hoisted by the workshop crane via the equipped wireless infrared remote. Control the bracket mechanisms to rise to the specified height. Then, place the aircraft fuselage slowly on the bracket mechanisms of the platform. Drive the platform into station No. 0, and park it at the position where both cameras can capture the QR codes on the floor.
(b) Carry out the assembly jobs. After the platform arrives at the designated position, the wheels will brake automatically. The parking mechanisms will move synchronously to raise the platform and to bear the weight of the aircraft and the platform. Then, the assembly jobs can be carried out on the platform. During the assembly process, the aircraft can be adjusted to the specified height and be stably supported.
(c) Transport the aircraft to the next station. Move the parking mechanisms back to the original position synchronously through the control software. Enter the number of the target station and select the motion mode. Confirm that the navigation parameters are correct and click the start button. Then, the platform will automatically transport the aircraft to the target station and park in the design posture.

## 3. Navigation and Localization Study

### 3.1. Navigation Mathematical Model

**Figure 5:**
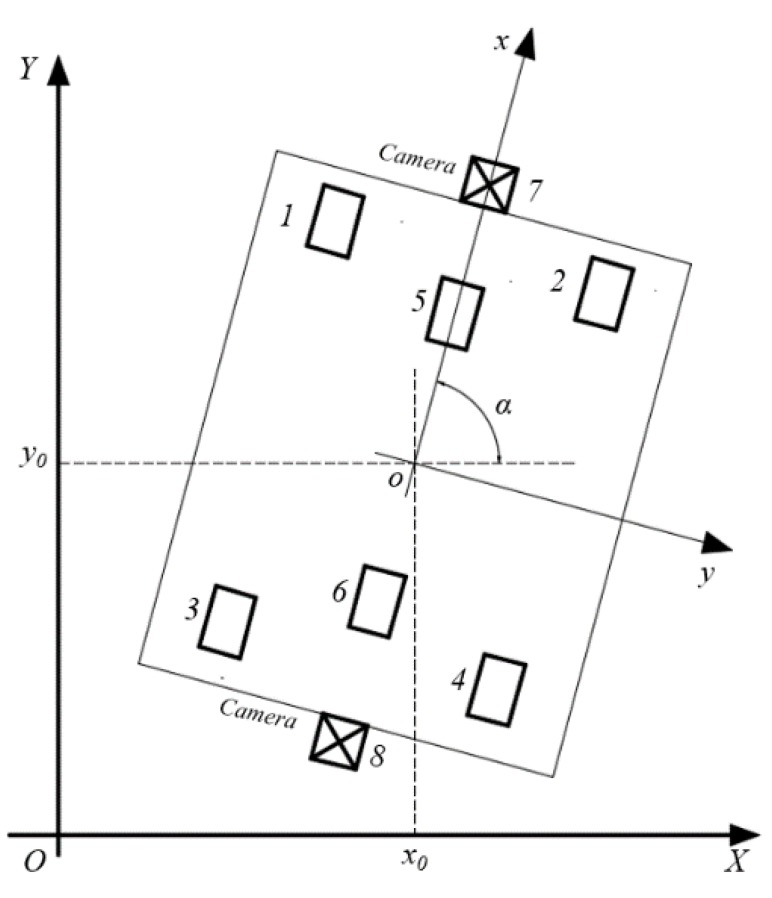
Global and local coordinate systems.

If the unevenness of the workshop floor is neglected and the transport platform is regarded as a rigid body, the motion of the platform in the workshop can be regarded as the plane motion of rigid bodies. The local coordinate system (x,o,y) of the transport platform is established by taking the geometric center of the platform as the origin and the forward direction as the positive direction of the x axis, as shown in Figure 5. In Figure 5, wheel No. 1–4 are wheels for steering and No. 5–6 are drive wheels. Four industrial casters located in the middle of the platform only play an auxiliary supporting role and do not affect the motion of the platform, so they have been neglected in the model. The transformation equation between local and global coordinates is as follows:

(1) $$\left[\begin{array}{c} X_{i}\\ Y_{i}\\ 1 \end{array}\right]=\left[\begin{array}{ccc} \cos\alpha & \sin\alpha & x_{0}\\ -\sin\alpha & \cos\alpha & y_{0}\\ 0 & 0 & 1 \end{array}\right]\left[\begin{array}{c} x_{i}\\ y_{i}\\ 1 \end{array}\right]$$

where $x_{0},y_{0}$ is the global coordinates of the origin of the local coordinate system, α is the angle between the local coordinate system and the global coordinate system, and $\left(x_{i},y_{i}\right)$ and $\left(X_{i},Y_{i}\right)$ (i=1,2,3…8) are the local and global coordinates of the wheels or cameras. Table 2 describes the local coordinates of the platform components designed in this work.

The position and angle of the platform are expressed by $\left[x_{0},\;y_{0},\;\alpha_{0}\right]$ which are all functions of time. The motion of the platform can be modelled as follows:

(2) $$x_{0}(t)=f_{1}(t),\;y_{0}(t)=f_{2}(t),\;\alpha_{0}(t)=f_{3}(t)$$

The motion of the platform between stations can be regarded as the process from the initial position and posture $\left[x_{0},\;y_{0},\;\alpha_{0}\right]$ of a certain station to the target position and posture $\left[x_{0}^{\prime },\;y_{0}^{\prime },\;\alpha_{0}^{\prime }\right]$ of the next station through a certain motion mode in time *t*. The motion mode of the platform in this paper includes self-rotational motion, linear motion, and fixed-axis rotation.

#### 3.1.1. Self-Rotational Motion

The self-rotational motion is used to adjust the forward direction of the platform, which is achieved by turning the six wheels to 90 ° and by giving the two drive wheels opposite speeds. The angle of the rotation can be controlled by the distance of the drive wheels, which can be obtained by the following:

(3) $$\Delta \alpha =\frac{S}{R}=\frac{S}{y_{5}}$$

where Δα is the angle of rotation, which is positive for clockwise motion and negative for counterclockwise motion, and where S is the distance of the drive wheels.

#### 3.1.2. Linear Motion

**Figure 6:**
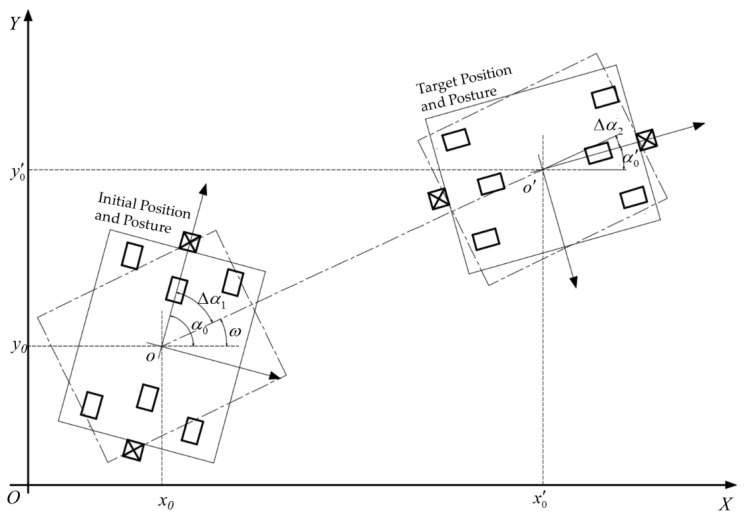
Navigation process in linear motion mode.

Linear motion is the most used motion mode. Its process includes three phases: self-rotation to adjust the direction, linear motion to reach the target position, and self-rotation to reach the target posture, as shown in Figure 6.

Since both the first and the third phases are self-rotating motions, the distances $S_{1}$ and $S_{3}$ of the drive wheels can be calculated by Equation (3). The corresponding rotation angles $\Delta \alpha_{1}$ and $\Delta \alpha_{2}$ can be obtained from the angle ω between the straight line $oo^{\prime }$ and the X axis, which is as follows:

(4) $$\begin{array}{cc} \Delta \alpha_{1}=\alpha_{0}-\omega & \Delta \alpha_{2}=\omega - \end{array}\alpha_{0}^{\prime }$$

where ω can be obtained from the start and end positions of the platform:

(5) $$\omega =\left\{ \begin{array}{l} -\begin{array}{cc} \frac{\pi }{2} & x_{0}^{\prime }=x_{0},y_{0}^{\prime }<y_{0} \end{array}\\ \begin{array}{cc} \frac{\pi }{2} & x_{0}^{\prime }=x_{0},y_{0}^{\prime }>y_{0} \end{array}\\ \begin{array}{cc} \arctan\frac{y_{0}^{\prime }-y_{0}}{x_{0}^{\prime }-x_{0}} & x_{0}^{\prime }\neq x_{0} \end{array} \end{array}\right.\omega \in \left[-\frac{\pi }{2},\frac{\pi }{2}\right]$$

The distance $S_{2}$ of the second phase can be calculated by the distance formula between two points. Apply the coordinates of the initial and target positions to the distance formula, which is as follows:

(6) $$S_{2}=\sqrt{{\left(y_{0}^{\prime }-y_{0}\right)}^{2}+{\left(x_{0}^{\prime }-x_{0}\right)}^{2}}$$

#### 3.1.3. Fixed-Axis Rotation

**Figure 7:**
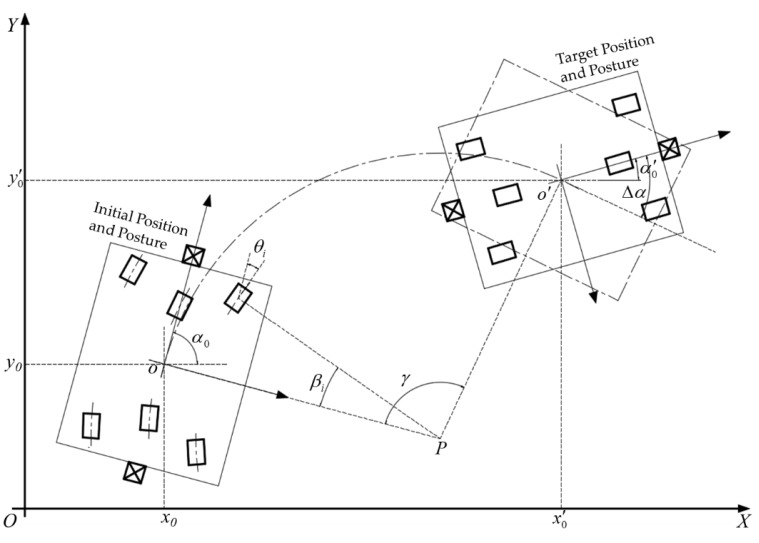
Navigation process in fixed-axis rotation mode.

Figure 7 presents the two phases of the fixed-axis rotation, which is rotation around a fixed axis to the target position and self-rotation to the target posture.

The path of the first phase is an arc with the start position and the target position as the two endpoints and the heading of the initial posture as the tangent direction. Therefore, it is required that the target position point is not on the initial heading direction line, which can be expressed as follows:

(7) $$y_{0}^{\prime }-y_{0}\neq \left(x_{0}^{\prime }-x_{0}\right)\tan\alpha_{0}$$

Assuming that the global coordinates of the center P of the arc path are $\left(X_{P},Y_{P}\right)$, then $\left|oP\right|=\left|o^{\prime }P\right|$ can be described as follows:

(8) $${\left(x_{0}-X_{P}\right)}^{2}+{\left(y_{0}-Y_{P}\right)}^{2}={\left(x_{0}^{\prime }-X_{P}\right)}^{2}+{\left(y_{0}^{\prime }-Y_{P}\right)}^{2}$$

P is on the line oP, which means the following:

(9) $$Y_{P}=X_{P}\cot\alpha_{0}+y_{0}-x_{0}\cot\alpha_{0}$$

Based on Equations (8) and (9), we can calculate the global coordinates $\left(X_{P},Y_{P}\right)$ of the center P. Then, we plug $\left(X_{P},Y_{P}\right)$ into Equation (1) and we can obtain the local coordinates $\left(x_{P},y_{P}\right)$ of the center P. Let $\theta_{i}$ denote the angle need to rotate of No. *i* wheel, (i=1,2,3…6). According to the geometrical properties of a circle, $\theta_{i}$ is equal to the corresponding central angle $\beta_{i}$, which can be calculated by the following:

(10) $$\begin{array}{c} \tan\theta_{i}=\tan\beta_{i}=\frac{y_{i}}{x_{p}-x_{i}},\\ \theta_{i}=\begin{array}{cc} \arctan\frac{y_{i}}{x_{p}-x_{i}} & \theta_{i}\in \left(-\frac{\pi }{2},\frac{\pi }{2}\right) \end{array} \end{array}$$

where $\theta_{i}$ is also positive for clockwise motion and negative for counterclockwise motion.

It is assumed that the central angle of the arc path is γ, and then, the distance of the first phase can be obtained by the following:

(11) $$S_{1}=\sqrt{{\left(y_{5}-y_{p}\right)}^{2}+{\left(x_{5}-x_{p}\right)}^{2}}\times \gamma$$

where γ is equal to the angle between the line oP and the line $o^{\prime }P$. γ also depends on which side of the initial orientation the target position is located on, which means γ needs to be expressed in the form of piecewise functions:

(12) $$\gamma =\left\{ \begin{array}{l} \begin{array}{cc} \frac{\pi }{2}+\alpha_{0}-\arctan\frac{y_{0}^{\prime }-Y_{p}}{x_{0}^{\prime }-X_{P}} & y_{0}^{\prime }-y_{0}<\left(x_{0}^{\prime }-x_{0}\right)\tan\alpha_{0} \end{array}\\ \begin{array}{cc} \frac{\pi }{2}-\alpha_{0}+\arctan\frac{y_{0}^{\prime }-Y_{p}}{x_{0}^{\prime }-X_{P}} & y_{0}^{\prime }-y_{0}>\left(x_{0}^{\prime }-x_{0}\right)\tan\alpha_{0} \end{array} \end{array}\right.$$

The second phase of the fixed-axis rotation is a self-rotating motion, and the distance can be calculated by Equation (3). Correspondingly, the Δα term in this case is related to γ, the initial angle $\alpha_{0}$, and the target angle $\alpha_{0}^{\prime }$. According to the geometric relationships, Δα can be obtained by the following:

(13) $$\Delta \alpha =\left\{ \begin{array}{l} \begin{array}{cc} \alpha_{0}-\gamma -\alpha_{0}^{\prime } & y_{0}^{\prime }-y_{0}<\left(x_{0}^{\prime }-x_{0}\right)\tan\alpha_{0} \end{array}\\ \begin{array}{cc} \alpha_{0}+\gamma -\alpha_{0}^{\prime } & y_{0}^{\prime }-y_{0}>\left(x_{0}^{\prime }-x_{0}\right)\tan\alpha_{0} \end{array} \end{array}\right.$$

### 3.2. Localization Method

In theory, the platform will park at the assembly station in the design posture, which means that the QR code will be in the center of the image captured by the CMOS camera. However, in actual navigation, because of the existence of motion errors, the actual position and posture will deviate from the theoretical values, which leads to a deviation between the QR code and the image center. As the cameras are fixed on the platform, there must be a quantitative relationship between the two deviations which can help us to obtain the deviation of the platform from the images and locate the platform precisely.

#### 3.2.1. Image Acquisition

**Figure 8:**
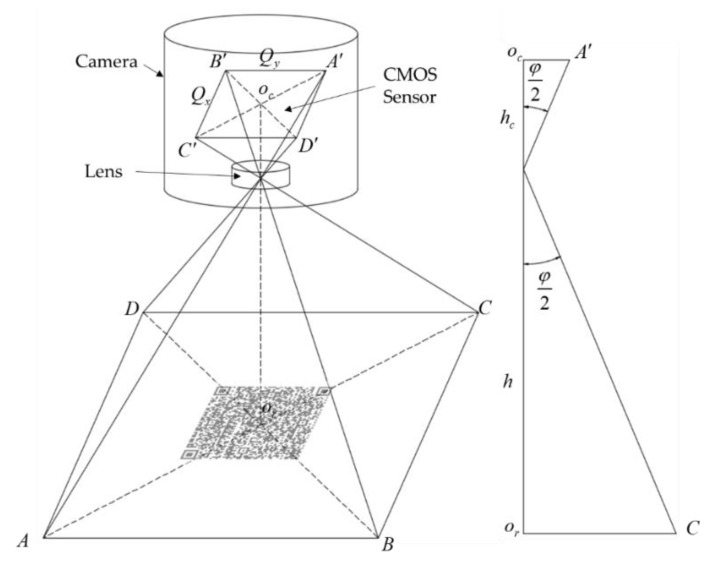
Simplified camera image acquisition model.

To measure distance and angle deviation of the platform through the QR code images, it is necessary to explore the correspondence of the image with the captured scene. Figure 8 illustrates a simplified camera image acquisition model with the camera components in detail. 

Let the length of each pixel be λ mm. According to the relationship between the real scene distance and the image distance shown in Figure 8, we can calculate λ by the triangle similarity theorems:

(14) $$\Delta \alpha =\left\{ \begin{array}{l} \begin{array}{cc} \alpha_{0}-\gamma -\alpha_{0}^{\prime } & y_{0}^{\prime }-y_{0}<\left(x_{0}^{\prime }-x_{0}\right)\tan\alpha_{0} \end{array}\\ \begin{array}{cc} \alpha_{0}+\gamma -\alpha_{0}^{\prime } & y_{0}^{\prime }-y_{0}>\left(x_{0}^{\prime }-x_{0}\right)\tan\alpha_{0} \end{array} \end{array}\right.$$

where φ is the field of view angle, W,L is the CMOS size, f is the focal length, $Q_{x},Q_{y}$ is the image resolution, and h is the installation height defined by the platform project. Table 3 describes the parameters of the camera used in this work.

#### 3.2.2. Image Processing

**Figure 9:**
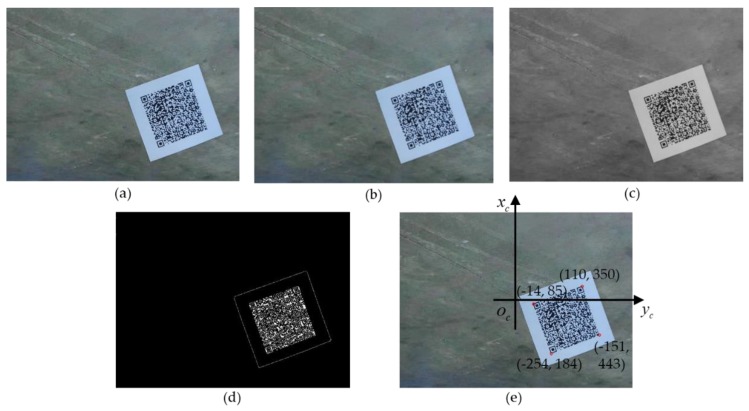
The processing resultant images: (**a**) original image; (**b**) after a Gauss filter; (**c**) converted to gray-scale; (**d**) after Canny edge detection; and (**e**) the original image with the image coordinate system and the coordinates of the four corners of the QR code.

The origin images captured by the cameras need to be denoised and preprocessed to posterior information extraction, as shown as an example in Figure 9a. The image processing algorithm initiates with a Gaussian filter to remove salt-and-pepper noise and to smooth the images. Equation (15) gives the two-dimensional Gaussian function:

(15) $$G\left(x,y\right)=\frac{1}{2\pi \sigma^{2}}\exp\left(-\frac{x^{2}+y^{2}}{2\sigma^{2}}\right)$$

where σ is the standard deviation that controls the smoothness of the image. The smaller the value, the higher the positioning accuracy of the filter but the lower the signal-to-noise ratio. On the contrary, a higher σ value leads to a lower positioning accuracy but a higher signal-to-noise ratio. After many tests, the value of σ in this paper is set to 2, and the filtered image is shown in Figure 9b.

For the RGB (Red, Green, and Blue) color image, each pixel contains three components and each component has 256 values [23], which results in a large amount of computation. In order to reduce the computational complexity, the filtered image is converted to a grayscale image which contains only brightness information. Figure 9c gives the converted image.

In order to describe the accurate position of the QR code in the image, the image coordinate system $\left(x_{c},o_{c},y_{c}\right)$ is established by taking the center of the image as the origin and the forward direction as the positive direction of x axis, as shown in Figure 9e. Canny edge detection is applied to obtain the coordinates of the edges in the image. Canny edge detection algorithm is a classical edge detection algorithm based on image gradient calculation developed by John F. Canny in 1986 [24,25]. Figure 9d is the binary image with clear Canny edge in white and background in black.

Since the QR code is a standard square, it is easy to obtain its position by the coordinates of its four corners. Based on Canny edge detection results, coordinates of four corners of the QR code are extracted, as shown in Figure 9e. Then, we can use the four coordinates to calculate the position of the QR code and to measure the distance and angle deviation of the platform.

#### 3.2.3. Deviation Calculation

For ease of description, let $\left(x_{A},y_{A}\right)$, $\left(x_{B},y_{B}\right)$, $\left(x_{C},y_{C}\right)$, and $\left(x_{D},y_{D}\right)$ denote the coordinates of the four corners obtained by the abovementioned image processing. In this paper, the coordinates of the center point $\left(x_{qr},y_{qr}\right)$ of the QR code are used to represent its position. For reducing the error of edge detection, the four-point average method is used to calculate the coordinates of the center point:

(16) $$x_{qr}=\frac{x_{A}+x_{B}+x_{C}+x_{D}}{4},\;y_{qr}=\frac{y_{A}+y_{B}+y_{C}+y_{D}}{4}$$

In order to calculate the deviation of the platform, it is necessary to convert the coordinates in the image coordinate system $\left(x_{c},o_{c},y_{c}\right)$ into the current platform coordinate system $\left(x_{r},o_{r},y_{r}\right)$. Based on Equation (15), the length of each pixel λ and the local coordinates of the front camera $\left(x_{7},y_{7}\right)$ and back camera $\left(x_{8},y_{8}\right)$, we can derive the transformation equation between the two coordinate systems:

(17) $$\begin{array}{cc} \left[\begin{array}{c} x_{r}\\ y_{r}\\ 1 \end{array}\right]=\left[\begin{array}{ccc} \lambda & 0 & x_{i}\\ 0 & \lambda & y_{i}\\ 0 & 0 & 1 \end{array}\right]\left[\begin{array}{c} x_{c}\\ y_{c}\\ 1 \end{array}\right] & \left(i=7,8\right) \end{array}$$

**Figure 10:**
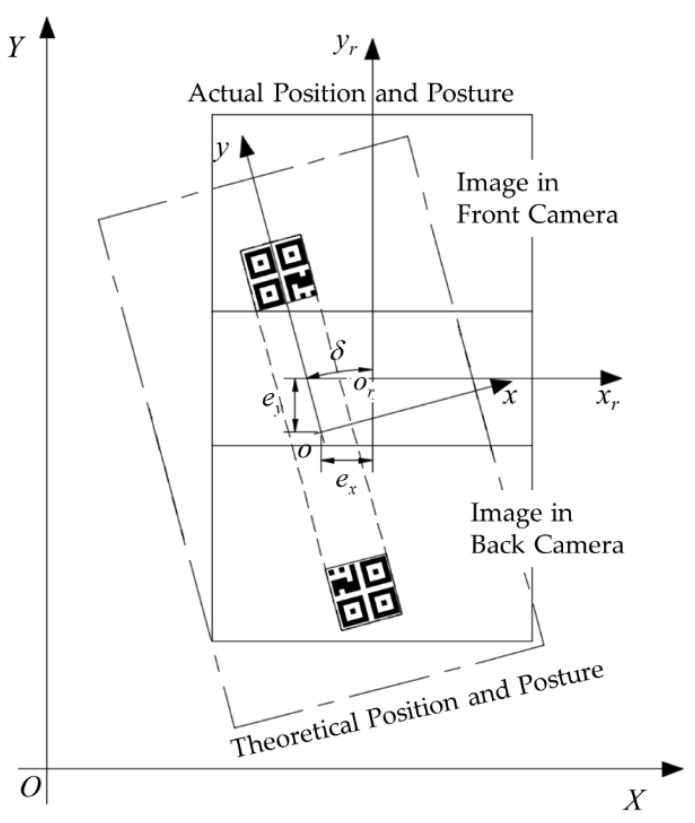
Schematic of the quick response (QR) code images in the two cameras when the platform is parked at a certain assembly station.

Figure 10 presents the QR code images in the front and rear cameras when the platform is parked at a certain assembly station. The actual position and posture of the platform $\left[x_{r0},\;y_{r0},\;\alpha_{r0}\right]$ can be modelled as follows:

(18) $$\begin{array}{ccc} x_{r0}=x_{0}+e_{x} & y_{r0}=y_{0}+e_{y} & \alpha_{r0}=\alpha_{0}+\delta \end{array}$$

where δ is the angle deviation, and where $e_{x}$ and $e_{y}$ are the distance deviations in the *x* and *y* directions, respectively.

Let $q_{r1}$ and $q_{r2}$ denote the center points of the QR code in the front and back cameras, respectively. According to Figure 10, the slope of the connection between $q_{r1}$ and $q_{r2}$ is equal to the angle deviation δ, and the midpoint of $q_{r1}$ and $q_{r2}$ is the theoretical position of the platform in the current platform coordinate system $\left(x_{r},o_{r},y_{r}\right)$. Based on this relationship, we can calculate the angle deviation δ and the distance deviation $e_{x}$ and $e_{y}$ through the coordinates of these two points in the current platform coordinate system. Let $\left(x_{qr1},y_{qr1}\right)$ and $\left(x_{qr2},y_{qr2}\right)$ denote the coordinates of the two center points in their image coordinate systems, which can be obtained by Equation (16). Then, plug $\left(x_{qr1},y_{qr1}\right)$ and $\left(x_{qr2},y_{qr2}\right)$ into Equation (17) and we can obtain the coordinates of the two points in the current platform coordinate system, which are $\left(\lambda x_{qr1}+\frac{L}{2},\lambda y_{qr1}\right)$ and $\left(\lambda x_{qr2}-\frac{L}{2},\lambda y_{qr2}\right)$, respectively. By substituting the two coordinates into the mentioned geometric relationships, the final deviation calculation formula is derived as follows:

(19) $$\begin{array}{c} \delta =\begin{array}{cc} \arctan\frac{y_{qr1}-y_{qr2}}{x_{qr1}-x_{qr2}+L/\lambda } & \delta \in \left(-\frac{\pi }{2},\frac{\pi }{2}\right) \end{array}\\ e_{x}=\left(-\frac{y_{qr1}+y_{qr2}}{2}\times \sin\delta -\frac{x_{qr1}+x_{qr2}}{2}\times \cos\delta \right)\times \lambda \\ e_{y}=\left(\frac{x_{qr1}+x_{qr2}}{2}\times \sin\delta -\frac{y_{qr1}+y_{qr2}}{2}\times \cos\delta \right)\times \lambda \end{array}$$

The actual position and posture of the platform can be obtained by substituting the results of Equation (19) into Equation (18). Based on the corrected position and posture, the input target station number, and the selected motion mode, the computer can calculate the accurate navigation path and generate the required motor commands for the wheels to drive the platform to the target station. 

## 4. Experimentation

**Figure 11:**
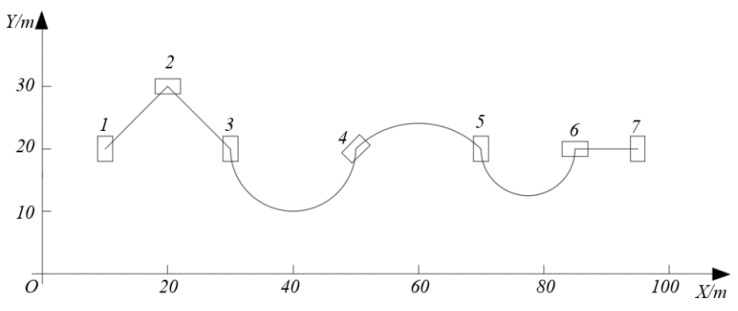
Experimental path schematic.

To verify the feasibility and effectiveness of the navigation and localization method, experiments were performed on a design path containing seven stations. Figure 11 shows the experimental path schematic. The design position and posture of each station were stored in the computer and sorted by the station numbers. The station numbers were encoded into the QR codes, which were placed on the floor for the cameras to read.

**Figure 12:**
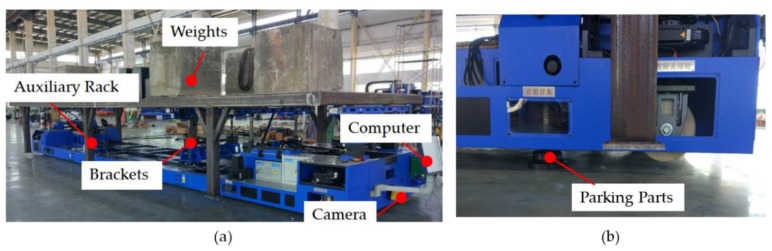

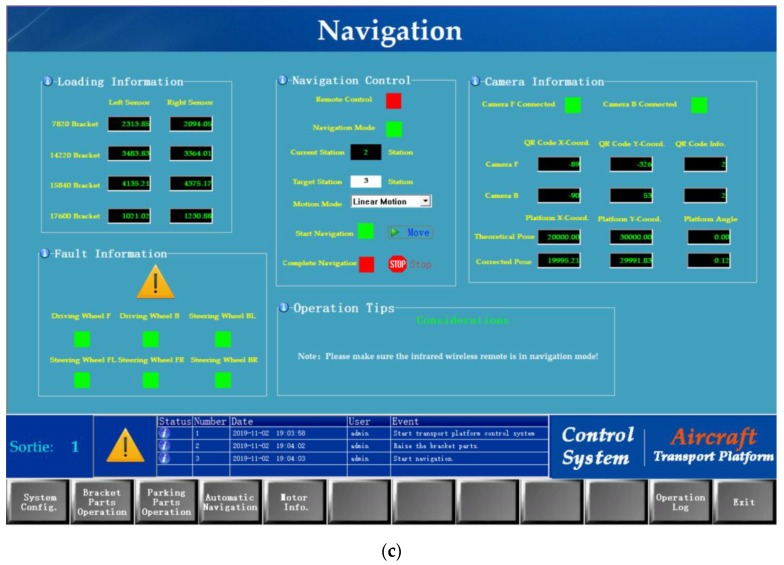
Navigation experiments in a workshop: (**a**) weights distribution; (**b**) parking situation; and (**c**) software interface.

To reproduce the condition closest to the actual working conditions, the platform was loaded with weights by using an auxiliary rack. The weight of the weights was slightly heavier than the target aircraft, and the weights were distributed according to the weight distribution of the target aircraft. In this paper, the total weight of the loading weights was 22 T, as shown in Figure 12a. After loading, the platform was driven to station No. 1 of the test path by the equipped wireless infrared remote and parked at the position where both of the front and back QR codes can be captured by the two cameras, as shown in Figure 12b. Then, the remote control was switched to the navigation mode. Through the control software, according to the design path, station No. 2 was input as the target station and linear motion was selected as the motion mode. The subsequent station number and motion mode were input into the control software when the platform arrived at the target station. In addition to the user input, the software interface, as shown in Figure 12c, also displays real-time loading information of the four bracket mechanisms, fault information of the wheels, and the QR code information obtained by the cameras, which is helpful for the user to monitor the status of the platform in the navigation process.

When the platform was parking at each station, the actual position and angle were measured and recorded and the deviation between the actual and theoretical values was calculated. The results of the first navigation test are summarized in Table 4. From Table 4, one can observe that the deviation of station one is much larger than that of other stations. This is because the platform is driven by the wireless infrared remote into station one, which makes it difficult to adjust the position and posture accurately. Still, at station two, the position deviation is reduced to within 10 mm and the angle deviation is reduced to 0.13°, which means that the transport platform can obtain the correct path to the second station and can drive into the second station accurately. It is verified that, through our algorithm, the theoretical position and the posture of station one were corrected to the actual values. From the deviation of subsequent stations, it can be summarized that, in the first navigation experiment, the maximum distance deviation in the X direction is −5.87 mm, the maximum *Y*-directional deviation is −8.89 mm, and the maximum angle deviation is −0.142°, which preliminary demonstrates that the proposed method is a feasible and effective solution for aircraft transportation between assembly stations.

**Figure 13:**
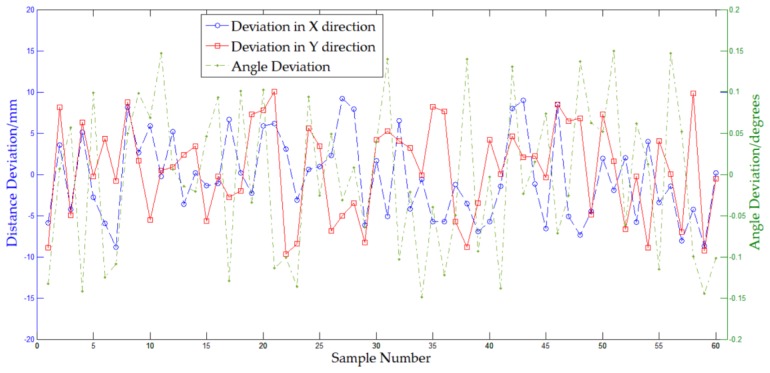
Distance and angle deviations of the repeated tests.

In order to analyze the accuracy of the transport platform, repeated tests were carried out. Likewise, the position and posture of the platform at each station were measured and recorded. As cited, the data of station one was independent of the navigation accuracy and was removed in the analysis. As a summary of trials results, Figure 13 displays a line chart of the calculated position and posture deviation of the other stations. In Figure 13, one can visually perceive the overall performance of the platform. The platform runs stably, and the deviation is randomly distributed within a certain limit.

Compared with the positive and negative directions of the deviation, the value is more important for the accuracy of the platform. Therefore, we used the absolute deviation of the test results for quantitative analysis. Table 5 summarizes the statistics analysis of the processed data. Observing Table 5, the deviations in the X and Y directions are basically the same, which indicates that the distance deviation of the platform in this paper does not have directionality. Thus, the average of the deviations in the two directions was considered as the overall distance deviation of the platform, as shown in the third line of Table 5. Generally, the position deviation of the platform is less than 10 mm, with 4.57 mm on average, while the angle deviation is less than 0.15°, with 0.079° on average. The acceptable position and angle deviations of the aircraft at each assembly station are mainly determined by the size of the aircraft, and in this paper, they need to be within ±25 mm and ±0.2° respectively. Therefore, the platform can meet the position and posture requirements of the target aircraft at the assembly station in this paper.

To explore the relationship between the deviation and motion mode, the experimental data were divided into two groups: one was linear motion, while the other was fixed-axis rotation. Therefore, data of two, three, and seven stations and four, five, and six stations were extracted and analyzed, respectively. Table 6 summarizes and compares the two groups data. From Table 6, it can be observed that, both on average and at maximum, the distance deviation in linear motion mode is better than that in fixed-axis rotation while the angle deviation is almost the same. It might be interpreted that the distance deviation is mainly the result from the navigation route, which is more complicate in fixed-axis rotation mode, and that the angle deviation is caused by the final angle adjustment, which is the same in the two motion modes. Thus, the platform in this work has a better position accuracy in linear motion mode than in fixed-axis rotation mode.

## 5. Conclusions

In this paper, an efficient and feasible navigation and localization method has been proposed for the transport platform for aircraft final assembly. The guiding approach is based on two CMOS cameras installed on the front and back of the platform. The platform obtains the theoretical position information by decoding the QR code images captured by the two cameras. Compared with other navigation methods in literature, the proposed approach has some advantages in terms of the cost of installation and changing routes. To obtain the precise position and angle of the platform, a localization method based on the QR code images is proposed. Canny edge detection is applied to the processed image to obtain the position of the QR code in the image. By constructing the relationship between the captured real scene and the image, the angle/distance deviation of the platform can be obtained by the image. Then, the actual position and posture of the platform can be calculated based on the obtained deviation. The proposed method has been integrated into the platform and has been tested on the design path in a workshop. The results suggest that the position accuracy of the platform is within ±10 mm and that the angle error is within ±0.15°, which can meet the position and posture requirements of the target aircraft at the assembly station in this paper. 

As future work, in terms of error reduction, the work points in two directions. The first point concerns the fixed-axis rotation. As discussed, the performance of the fixed-axis rotation mode is worse than that of the linear motion mode. It suggests that we can explore a simpler control strategy for the fixed-axis rotation mode. The other direction refers to the sensor and image which affect the accuracy of localization. In this sense, in the future, we plan to investigate the relationship between the parameters of CMOS sensor camera and the deviation of the platform and to improve the edge detection algorithm according to the characteristics of the acquired images.

## Figures and Tables

**Table 1:** Dimensions and specifications of the transport platform.

Description	Quantity
Length × Width × Height	10,500 × 2500 × 1300 mm
Mass	10.2 kg
Max. Linear Velocity	1.67 m/s
Drive Wheel Diameter	410 mm
Diameter of the Wheel for Steering	300 mm

**Table 2:** Local coordinates of the wheels and cameras.

No.	Components	Local Coordinates	No.	Components	Local Coordinates
1	FL Wheel	(4970, −925)	5	Front Drive Wheel	(4815, 0)
2	FR Wheel	(4970, 925)	6	Back Drive Wheel	(−4815, 0)
3	BL Wheel	(−4970, −925)	7	Front CMOS Camera	(5346, 0)
4	BR Wheel	(−4970, 925)	8	Back CMOS Camera	(−5346, 0)

Note: FL, FR, BL and BR are short for front left, front right, back left and back right respectively. CMOS is short for complementary metal oxide semiconductor.

**Table 3:** Parameters description and values of the cameras used in this work.

Description	Quantity
Brand Model	VISIONGO SGIB1280-60 gm/gc
Sensor Technology	CMOS image sensor
Max. Frame Rate	60 fps
Pixel Resolution	1280 × 1024 pixels
CMOS Size	7.716 × 5.319 mm
Focal Length	20 mm
Installation Height	455 mm

**Table 4:** Theoretical and actual positions and postures at each station of the first test.

No.	Theoretical Value (mm, mm, °)	Actual Value (mm, mm, °)	Deviation (mm, mm, °)
1	(10,000.00, 20,000.00, 90.000)	(10,051.23, 20,031.36, 88.620)	(51.23, 31.36, −1.380)
2	(20,000.00, 30,000.00, 0.000)	(19,994.13, 29,991.11, −0.132)	(−5.87, −8.89, −0.132)
3	(30,000.00, 20,000.00, −90.000)	(30,003.53, 20,008.11, −89.994)	(3.53, 8.11, 0.006)
4	(50,000.00, 20,000.00, 45.000)	(49,995.73, 19,995.01, 45.056)	(−4.27, −4.99, 0.056)
5	(70,000.00, 20,000.00, −90.000)	(70,005.14, 20,006.32, −89.858)	(5.14, 6.32, −0.142)
6	(85,000.00, 20,000.00, 0.000)	(84,997.24, 19,999.76, 0.099)	(−2.76, −0.24,0.099)
7	(95,000.00, 20,000.00, 90.000)	(94,994.02, 20,004.35, 89.875)	(−5.98, 4.35, −0.125)

**Table 5:** Total trial data summary for the repeated tests.

Statistical Items	Average Value	Max. Value
Deviation in X direction	4.82 mm	9.21 mm
Deviation in Y direction	4.32 mm	9.99 mm
Distance Deviation	4.57 mm	9.99 mm
Angle Deviation	0.079°	0.149°

**Table 6:** Comparison between the linear motion and the fixed-axis rotation.

Statistical Items	Distance Deviation Mean	Distance Deviation Max.	Angle Deviation Mean	Angle Deviation Max.
Linear Motion	4.43 mm	8.98 mm	0.085°	0.147°
Fixed-axis Rotation	4.72 mm	9.99 mm	0.084°	0.149°
Difference	0.29 mm	1.01 mm	0.001°	0.002°
Proportion	6.54%	11.2%	1.17%	1.36%

## References

[1] Raju J. A Conceptual design and cost optimization methodology. Proceedings of the 44th AIAA/ASME/AHS Structures, Structural Dynamics and Materials Conference.

[2] Mas F., Ríos J., Menéndez J.L., Gómez A. (2013). A process-oriented approach to modeling the conceptual design of aircraft assembly lines. Int. J. Adv. Manuf. Technol..

[3] Pan Z., Guo Y., Zha S., Zhang S. (2018). Aircraft pulsating assembly line balancing problem based on hybrid algorithm. Comput. Integr. Manuf. Syst..

[4] Ren Y., Lu Z. (2019). A flexible resource investment problem based on project splitting for aircraft moving assembly line. Assem. Autom..

[5] Barbosa G.F., Hernandes A.C., Luz S., Batista J. (2017). A conceptual study towards delivery of consumable materials to aircraft assembly stations performed by mobile robot based on industry 4.0 principles. Int. J. Aeronaut. Aerosp. Eng..

[6] Advanced Integration Technology: Boeing 787 Final Assembly. http://www.aint.com/projects/787_project/boeing_787_final_assembly.

[7] Lu Z., Zhu H., Han X., Hu X. (2018). Integrated Modelling and algorithm of material delivery and line-side storage for aircraft moving assembly lines. Int. J. Prod. Res..

[8] Caridá V., Morandin O., Tuma C. (2015). Approaches of fuzzy systems applied to an AGV dispatching system in a FMS. Int. J. Adv. Manuf. Technol..

[9] Galasso F., Rizzini D.L., Oleari F. (2019). Efficient calibration of four wheel industrial AGVs. Robot. Comput. Integr. Manuf..

[10] Fedorko G., Honus S., Salai R. (2017). Comparison of the Traditional and Autonomous AGV Systems. MATEC Web Conf..

[11] Zhang X., Huo L. (2017). A vision/inertia integrated positioning method using position and orientation matching. Math. Probl. Eng..

[12] Diogo P.O., Wallace P.N.R., Orides M.J. (2019). A qualitative analysis of a USB camera for AGV control. Sensors.

[13] Xing W., Chao S., Zou T. (2019). SVM-based image partitioning for vision recognition of AGV guide paths under complex illumination conditions. Robot. Comput. Integr. Manuf..

[14] Lee J., Hyun C.H., Park M. (2013). A vision-based automated guided vehicle system with marker recognition for indoor use. Sensors.

[15] Kim G., Petriu E.M. Fiducial Marker Indoor Localization with Artificial Neural Network. Proceedings of the IEEE/ASME International Conference on Advanced Intelligent Mechatronics (AIM).

[16] Petriu E.M., McMath W.S., Yeung S.K., Trif N., Bieseman T. (1993). Two-dimensional position recovery for a free-ranging automated guided vehicle. IEEE Trans. Instrum. Meas..

[17] Ryan H., Daniel O. (2014). Utilization of a vision system to automate mobile machine tools. SAE Int. J. Mater. Manuf..

[18] Li J., Wu Z., Zhang J. Research of AGV positioning based on the two-dimensional code recognition method. Proceedings of the 2015 International Conference on Logistics, Informatics and Service Sciences (LISS).

[19] Zhou C., Liu X. The study of applying the AGV navigation system based on two-dimensional bar code. Proceedings of the 2016 International Conference on Industrial Informatics-Computing Technology, Intelligent Technology, Industrial Information Integration (ICIICII).

[20] Rostkowska M., Topolski M. (2015). On the application of QR codes for robust self-localization of mobile robots in various application scenarios. Adv. Intell. Syst. Comput..

[21] Cavanini L., Cimini G., Ferracuti F. A QR code localization system for mobile robots: Application to smart wheelchairs. Proceedings of the European Conference on Mobile Robots.

[22] Nguyen T.T., Yong-Tae K. (2017). Navigation method of the transportation robot using fuzzy line tracking and QR code revognition. Int. J. Humanoid Robot..

[23] Nesson C. (2013). Encoding Multi-Layered Data into QR Codes for Increased Capacity and Security. Ph.D. Thesis.

[24] Canny J. (1986). A computational approach to edge detection. IEEE Trans. Pattern Anal. Mach. Intell..

[25] Pellegrino F.A., Vanzella W. (2004). Edge detection revisited. IEEE Trans. Syst. Man Cybern..

